# Correlation Between Mean Temperature and Incidence of Tick-borne Diseases Among Active Duty Service Members in the Contiguous U.S., 2000–2023

**Published:** 2025-03-20

**Authors:** Prabhavi Denagamage, Sithembile L. Mabila

**Affiliations:** 1 Defense Health Agency, Armed Forces Health Surveillance Division, Silver Spring, MD

## Abstract

Medical encounter data and reportable medical events from the Defense Medical Surveillance System were utilized to establish that from 2000 to 2023 there were 2,869 cases of Lyme disease and 175 cases of Rocky Mountain spotted fever among U.S. active component service members within the contiguous U.S. Coincident with a 5.3% (0.63°C) increase in annual mean temperature over the course of the 24-year surveillance period, annual Lyme disease incidence rates increased 35.5% overall, concurrently peaking with mean temperature in 2012 and 2016. After adjusting for annual mean, minimum and maximum temperatures, annual total precipitation, and regional climate, in addition to adjusting for age group, race and ethnicity, sex, and service, incidence rate ratios of both tick-borne diseases did not significantly change with increases in climatic variables. Adjusted incidence rate ratios of both tick-borne diseases increased with increasing age group.

**What are the new findings?:**

Annual Lyme disease incidence rates peaked concurrently with annual mean temperatures. Incidence rate ratios for Lyme disease were highest in the Southeast compared to the Northeast, while Rocky Mountain spotted fever incidence rate ratios were highest in the South compared to the Southeast. Annual Lyme disease and Rocky Mountain spotted fever incidence rates ratios increased with increasing age group.

**What is the impact on readiness and force health protection?:**

Incidence of both Lyme disease and Rocky Mountain spotted fever among U.S. active component service members may increase with worsening global warming. Evaluating how temperature affects these tick-borne diseases regionally helps identify protective measures for service members at risk.

## 1 BACKGROUND

Global warming can facilitate tick-borne disease (TBD) transmission through improved environmental favorability for ticks, extending their geographic range and periods of activity.^1^ Given that nearly 1.1 million U.S. active component service members (ACSMs) are stationed across the U.S., where several TBDs are endemic, it is crucial to understand how rising temperatures may influence the burden of such diseases.^2,3^

Evidence for the relationship between temperature and TBD in the U.S. is conflicting. Analysis of Lyme disease cases reported from 2000 to 2017 to the Centers for Disease Control and Prevention (CDC) determined that warming annual temperatures were associated with increasing incidence in the Northeast.^4^ A separate study used data from high-incidence states during 2000 to 2016 to predict that in subsequent decades, Lyme disease cases will increase by 21%, given the projected 2°C increase in annual mean temperature by mid-century.^5^ Meanwhile, a study utilizing data from the Kansas, Missouri, Arkansas, and Oklahoma state departments of health determined that average daytime land surface temperature above 35°C was a limiting factor for Rocky Mountain spotted fever (RMSF) incidence from 2005 to 2014.^6^ In further contrast, within the same surveillance period a cross-sectional study found that temperature was not associated with the presence or abundance of Lyme disease cases in southwest Virginia.^7^ Research on ACSMs in the eastern U.S. demonstrated a 5.7% increase in Lyme disease from 2006-2012, while another study has shown the Northeast has the highest incidence of Lyme disease from 2004 to 2013.^8,9^ Considering that minute changes in global warming can lead to considerable fluctuations in TBD burden, additional research must verify the relationship between temperature and TBD incidence, specifically among ACSMs, as there is no available literature on this topic specific to the U.S. Armed Forces.^10,11^

The objectives of this study were to identify the incidence of the 2 TBDs most frequently diagnosed within the Military Health System (MHS) among ACSMs and evaluate the correlation between temperature and incidence of each TBD, to inform pertinent public health professionals on control measures such as improvements to uniform permethrin treatment practices.^12,13^ Data available to the Armed Forces Health Surveillance Division (AFHSD), in conjunction with National Oceanic and Atmospheric Administration (NOAA) climatic data, may provide further clarity on the correlation between climate changes and TBD occurrence.

## 2 METHODS

This study included ACSMs from January 1, 2000 through December 31, 2023. Demographic and medical encounter data were obtained from the Defense Medical Surveillance System (DMSS) for confirmed cases of RMSF and Lyme disease acquired in the contiguous U.S. Diagnoses were ascertained from inpatient and outpatient encounter data, and reportable medical events (RMEs) of individuals who received medical care either in the MHS or civilian facilities in the purchased care system.

Adhering to the AFHSD surveillance case definition for RMSF, a case of RMSF was defined as 1 confirmed RME of RMSF or spotted fever rickettsiosis.^14^ Deviating from the AFHSD case definition, laboratory or epidemiological data were not included to confirm a RMSF case. As of January 1, 2010, RMEs of RMSF are expected to be reported as spotted fever rickettsioses, although this study identified RMEs of RMSF reported as RMSF after that date, through to 2017.^15^ Adhering to the AFHSD surveillance case definition for Lyme disease, a case of Lyme disease was defined as either 1 confirmed RME of Lyme disease, 1 inpatient encounter with a qualifying code in any diagnostic position, or 2 outpatient encounters within 60 days of one another with a qualifying code in any diagnostic position.^16^ Qualifying codes for Lyme disease were International Classification of Diseases, 9th Revision (ICD-9) code 088.81, or International Classification of Diseases, 10th Revision (ICD-10) codes beginning with A69.2.^16^

The first qualifying encounter or RME was deemed the incident encounter. ACSMs diagnosed with the TBD of interest before the surveillance period were excluded, and an individual could qualify as a Lyme disease or RMSF case only once. The location in which each TBD was acquired was determined to be the location of the facility in which the incident diagnosis was made. Demographic variables of interest were age, sex, race and ethnicity, service, and grade.

U.S. climate data on mean annual temperature and total annual precipitation were acquired from the National Oceanic and Atmospheric Administration (NOAA).^17^

The total numbers of each TBD were determined, and overall incidence rates for each TBD were calculated as diagnoses per 100,000 person-years (p-yrs) and stratified by age group, sex, race and ethnicity, service, and climate region (referred to as “regional climate” in this paper) as defined by NOAA.^17^ Annual incidence rates for Lyme disease and RMSF were also calculated as diagnoses per 100,000 p-yrs. The subgroup of each non-ordinal demographic variable with the most incident cases was selected as the reference group for adjusted incidence rate ratios (aIRRs). Poisson regression was used to calculate aIRRs (adjusted for annual mean temperature, annual minimum temperature, annual maximum temperature, annual total precipitation, regional climate, age group, race and ethnicity, sex, and service) and 95% confidence intervals (CIs) for each TBD. All analyses were conducted using SAS Enterprise Guide (version 8.3).

## 3 RESULTS

Among ACSMs in the contiguous U.S. from 2000 through 2023, there were 2,869 Lyme disease cases at a rate of 10.7 per 100,000 p-yrs, and 175 RMSF cases at a rate of 0.7 per 100,000 p-yrs (**Table 1**). Cases of both TBDs had a mean age of 31.3 years (standard deviation [SD]=8.7, range=18-61) (data not shown).

The highest incidence rates of Lyme disease (52.2 cases per 100,000 p-yrs) occurred in the Northeast while the highest rates of RMSF occurred in Ohio Valley (1.3 cases per 100,000 p-yrs) (**Table 1**). Lyme disease incidence rates increased with increasing age group, ranging from 7.0 cases per 100,000 p-yrs among ACSMs aged less than 20 years to 19.9 cases per 100,000 p-yrs among those aged 40 or more years (**Table 1**). Similarly, RMSF incidence rates increased with increasing age group, ranging from 0.4 cases per 100,000 p-yrs among ACSMs aged less than 20 years to 1.0 case per 100,000 p-yrs among those aged 40 or more years (**Table 1**). Lyme disease and RMSF incidence rates were highest among non-Hispanic White ACSMs, with this racial and ethnic group experiencing 13.0 Lyme disease cases per 100,000 p-yrs and 0.8 RMSF cases per 100,000 p-yrs (**Table 1**). Women had the highest rates of Lyme disease (14.0 cases per 100,000 p-yrs) while men had slightly higher rates of RMSF than women (0.7 cases per 100,000 p-yrs) (**Table 1**). Despite the Army having the greatest number of ACSMs compared to all other services, the incidence rates of Lyme disease were highest among Coast Guard members (28.6 cases per 100,000 p-yrs) and incidence rates of RMSF were highest among Marines (1.2 cases per 100,000 p-yrs) (**Table 1**).^2^

Overall annual mean temperature increased 5.3% (0.6°C) from 2000 to 2023, hitting a high of 12.9°C in 2012 and a low of 11.3°C in 2008 (**Figure 1a**). Mean annual temperatures were highest in the South and Southeast (**Figure 1a**). Overall total annual precipitation increased 4.5% (1.3 in.) over the surveillance period, hitting a high of 34.6 inches in 2018 and a low of 27.5 inches in 2012 (**Figure 1b**). Total annual precipitation was highest in the Northeast, Southeast, and Ohio Valley (**Figure 1b**).

**Figure 2:**
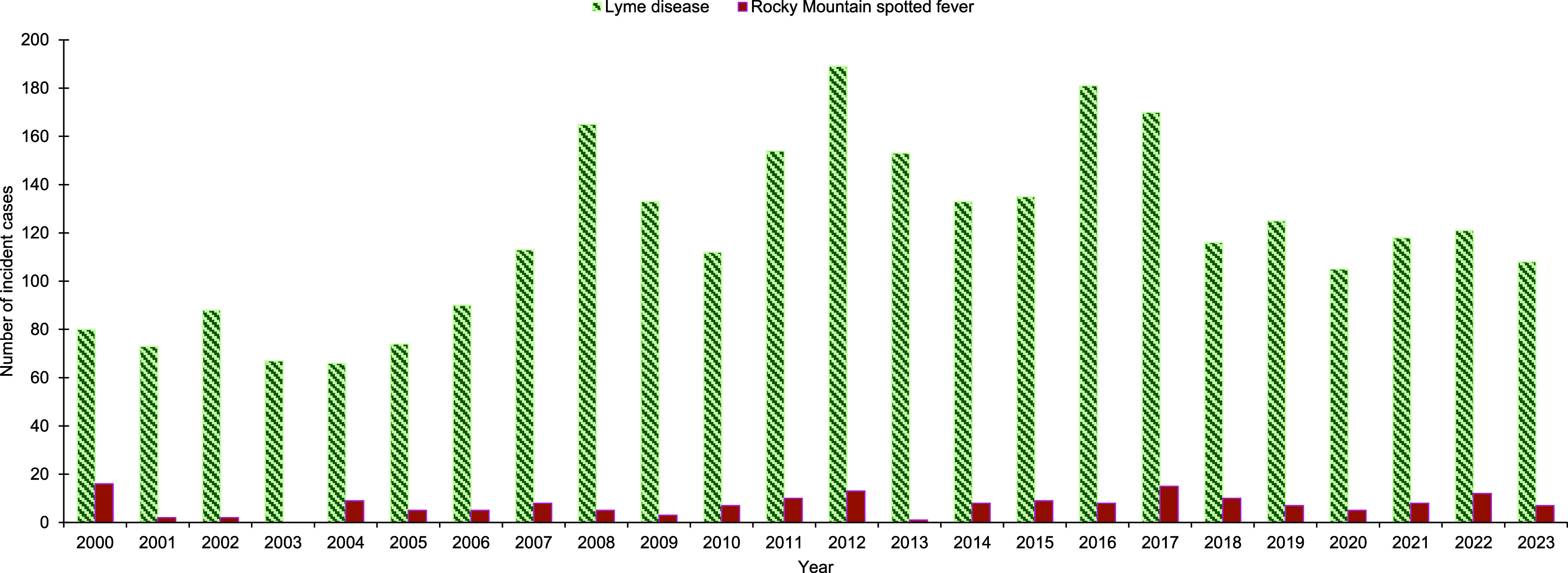
Annual Incidence of Lyme Disease and Rocky Mountain Spotted Fever, Active Component Service Members, Contiguous U.S., 2000–2023

Lyme disease incidence increased from 80 cases at a rate of 7.5 cases per 100,000 p-yrs in 2000 to 108 cases at a rate of 10.1 cases per 100,000 p-yrs in 2023 (**Figures 2**, **3a**). Over the 24-year surveillance period, the second highest peak in annual Lyme disease incidence rate (16.2 cases per 100,000 p-yrs) coincided with the highest annual mean temperature (12.9°C), highest annual maximum temperature (19.8°C), and lowest annual total precipitation (27.5 in.) (**Figures 1b**, **3a**). The highest annual Lyme disease incidence rate (18.2 cases per 100,000 p-yrs) coincided with the second-highest annual mean temperature (12.7°C), second highest annual maximum temperature (19.3°C), and highest annual minimum temperature (6.2°C) (**Figure 3a**). As mean temperature increased by 9.9% from 2011 to 2012, Lyme disease rates increased by 24.2%, and as mean temperature increased by 2.3% from 2015 to 2016, rates increased by 41.9% (**Figure 3a**). RMSF incidence decreased from 16 cases at a rate of 1.5 cases per 100,000 p-yrs in 2000 to 7 cases at a rate of 0.7 cases per 100,000 p-yrs in 2023, (**Figures 2**, **3b**). Rates were highest at 1.5 cases per 100,000 p-yrs in 2000 and 2017, followed by 1.1 cases per 100,000 p-yrs in 2012 (**Figure 3b**). As mean temperature increased by 9.9% from 2011 to 2012, RMSF rates increased by 32.1% (**Figure 3b**).

Throughout the surveillance period, annual Lyme disease incidence rates were consistently highest in the Northeast and intermittently highest in the Upper Midwest (**Figure 4a**). There was no clear trend in regional increases in annual RMSF incidence rates, however, the Southeast consistently had relatively high annual incidence rates over the surveillance period (**Figure 4b**). There were also noticeable spikes in incidence rates in Ohio Valley during 2005, 2008, 2017, 2020, and 2021 (**Figure 4b**).

After adjusting for all demographic variables of interest, for every 1°C increase in mean temperature and every 1 inch increase in total precipitation, the incidence rate of Lyme disease did not change (**Table 2**). For every 1°C increase in annual minimum temperature, the incidence rate ratio of RMSF increased by 20% (aIRR 1.2; 95% CI, 1.0-1.4) (**Table 2**). For every 1°C increase in annual mean temperature or 1 inch increase in total precipitation, the incidence rate ratio of RMSF did not change (**Table 2**).

The aIRR of Lyme disease was highest in the Southeast compared to the Northeast at 1.5 (95% CI, 1.3-1.6) (**Table 2**). When compared to the Southeast, there was no significant increase in aIRR for RMSF by regional climate (**Table 2**). The aIRRs of both TBDs increased with increasing age group compared to ACSMs less than 20 years of age, ranging from 1.0 (95% CI, 1.0-1.0) among ACSMs aged 20-24 years to 2.3 (95% CI, 2.3-2.4) among those aged 40 years or older for Lyme disease, and ranging from 1.0 (95% CI, 0.9-1.2) among ACSMs aged 20-24 years to 2.6 (95% CI, 2.3-3.0) among those aged 40 years or older for RMSF (**Table 2**). There was no significant increase in TBDs by race and ethnicity (**Table 2**). Women had the highest aIRR for Lyme disease compared to males, with this sex group having an aIRRs of 1.5 (95% CI, 1.5-1.5), while there was no significant difference in aIRRs by sex for RMSF (**Table 2**).

## 4 DISCUSSION

This study found that from 2000 to 2023 in the contiguous U.S., Lyme disease incidence rates increased 35.5% among ACSMs alongside a 5.3% (0.6°C) increase in overall annual mean temperature, meaning rates increased by 4.2 cases per 100,000 p-yrs for every 1°C increase in annual mean temperature. The CDC observed a more dramatic Lyme disease rate increase of 200% in the general U.S. population from 2000 to 2022, though it is important to note that in their analysis, suspect and probable cases were included starting in 2008, while this study included only confirmed cases.^18^

Conversely, RMSF incidence rates decreased 55.7% overall, or by 1.3 cases per 100,000 p-yrs for every 1°C increase in annual mean temperature, among ACSMs over the surveillance period. Although CDC data demonstrated an upward trend in incidence rates of confirmed and probable RMSF cases in the general U.S. population from 2000 to 2007, the proportion of confirmed cases over this period decreased from 15% to 4%.^19^

During the surveillance period, the highest annual Lyme disease incidence rate (18.2 cases per 100,000 p-yrs) coincided with the second highest annual mean temperature (12.7°C), while the second highest peak in annual Lyme disease incidence rate (16.2 cases per 100,000 p-yrs) coincided with the highest annual mean temperature (12.9°C) and lowest annual total precipitation (27.5 in.). Although previous research supports that increased Lyme disease incidence are correlated with warmer temperatures, the same data suggest that dry conditions reduce activity of the relevant tick vectors, which in turn lowers disease incidence.^4^

Over the 24-year surveillance period, RMSF incidence rates spiked at 1.1 cases per 100,000 p-yrs during the highest annual mean temperature (12.9°C) and lowest annual total precipitation (27.5 in.). Research has shown that increases in both average relative humidity and daytime land surface temperatures below 35°C increase RMSF incidence.^6^

Although crude Lyme disease rates were highest in the Northeast, adjusted results show that compared to the Northeast, rate ratios were highest in the Southeast. Although crude RMSF rates were highest in Ohio Valley, adjusted results show that compared to the Southeast, rate ratios were highest in the South. Surveillance of these TBDs has shown Lyme disease incidence rates among ACSMs were highest in the Northeast while spotted fever rickettsioses including RMSF are highly endemic to 5 states, 4 of which are in the South and Southeast.^9,20,21^

Crude rates as well as adjusted rate ratios of both TBDs increased with increasing age group. Women had 1.5 times the adjusted rate of Lyme disease than men but had an equal adjusted rate of RMSF compared to men. A previous study of ACSMs in the eastern U.S. also found that Lyme disease incidence rates increased with increasing age and women.^8^ Likewise, a 2000-2007 study of RMSF determined that incidence rates increased with increasing age group and cumulative incidence was higher among men than women.^19^

Crude rates and adjusted rate ratios were highest in the Coast Guard for Lyme disease and highest in the Marine Corps for RMSF. Many Coast Guard installations are concentrated in the Northeast, where Lyme disease is most incident.^20,22^ Similarly, one of the largest Marine Corps installations is located in North Carolina, which is a high incidence jurisdiction for RMSF.^21,23^ Furthermore, the location of Coast Guard and Marine Corps installations in coastal areas may explain the elevated incidence of TBD in each service, as rising sea levels may create environmental conditions that are more conducive to TBD transmission.^24,25^ Since the DMSS did not receive encounter data from the Coast Guard between 2015 and 2021, it is possible that Lyme disease and RMSF were even more incident in this service.^26^ Coast Guard combat uniforms are not factory treated with permethrin, which may also contribute to why ACSMs in this service experienced the highest aIRR of Lyme disease compared to Army members, whose combat uniforms are factory treated with permethrin.^13^

To our knowledge, this is the first study to investigate the relationship between climatic factors and TBD incidence in ACSMs, with a few limitations. First, the location of disease acquisition was deemed to be the location at which the incident encounter or RME occurred. Due to the range of possible incubation periods for each TBD, individuals may have developed symptoms and sought care in a location inconsistent with the location of infection.^27^ Therefore, the regional climate associated with ACSMs may be inaccurate. Likewise, the data do not clarify if the disease was acquired during or outside of work, making it difficult to ascertain a true correlation between TBD incidence and military activities. As a result, this may affect the control measures implemented to prevent infection within the Armed Forces as well as their efficacies. Furthermore, this study’s cohort may not include all cases, as Lyme disease and RMSF often present with nonspecific symptoms, which can make them difficult to diagnose.^27^ Lastly, the analysis does not account for anthropogenic factors such as land cover and host movement, which influence how climate affects TBD incidence.^28^

Although no significant correlation between temperature and TBD incidence was found, the observed peaks in annual Lyme disease incidence with rising mean temperatures reaffirm patterns in existing literature. Small changes in temperature can have substantial effects on TBD incidence, and these illnesses can reduce force readiness by causing fevers, headaches, and in some instances, neurologic and cardiac conditions.^10,27^ Further research is needed to explore the temporality of how climatic conditions affect incidence of both Lyme disease and RMSF. This information is essential to understand and improve the management of these illnesses among ACSMs.

## Figures and Tables

**Table 1:** Incidence Rates of Lyme Disease and Rocky Mountain Spotted Fever, by Demographic Variables, Active Component Service Members, Contiguous U.S., 2000–2023

	Lyme Disease			Rocky Mountain Spotted Fever		
	No.	Person-years	Rate^a^	No.	Person-years	Rate^a^
**Total**	2,869	26,797,177	10.7	175	26,797,177	0.7
**Regional climate**						
Northeast	1,161	2,222,856	52.2	13	2,222,856	0.6
Southeast	880	9,872,083	8.9	109	9,872,083	1.1
Northwest	51	1,538,504	3.3	1	1,538,504	0.1
Ohio Valley	244	2,050,622	11.9	26	2,050,622	1.3
Upper Midwest	13	98,075	13.3	0	98,075	0.0
South	280	4,748,720	5.9	20	4,748,720	0.4
Southwest	69	1,665,097	4.1	3	1,665,097	0.2
West	150	4,031,151	3.7	2	4,031,151	0.0
Northern Rockies and Plains	21	570,070	3.7	1	570,070	0.2
**Age group, y**						
<20	143	2,053,321	7.0	9	2,053,321	0.4
20-24	659	8,500,628	7.8	39	8,500,628	0.5
25-29	580	6,028,611	9.6	35	6,028,611	0.6
30-34	444	4,106,254	10.8	37	4,106,254	0.9
35-39	482	3,283,111	14.7	26	3,283,111	0.8
40+	561	2,825,252	19.9	29	2,825,252	1.0
**Racial and ethnic group**						
White, non-Hispanic	2,108	16,176,605	13.0	133	16,176,605	0.8
Black, non-Hispanic	257	4,363,708	5.9	13	4,363,708	0.3
Hispanic	254	3,474,928	7.3	17	3,474,928	0.5
Other/unknown^b^	250	2,781,936	9.0	12	2,781,936	0.4
**Sex**						
Male	2,290	22,659,259	10.1	151	22,659,259	0.7
Female	579	4,137,917	14.0	24	4,137,918	0.6
**Service branch**						
Army	1,085	9,500,810	11.4	59	9,500,810	0.6
Navy	649	6,695,784	9.7	41	6,695,784	0.6
Marine Corps	370	3,456,627	10.7	43	3,456,627	1.2
Air Force, Space Force	531	6,324,893	8.4	27	6,324,893	0.4
Coast Guard	234	819,063	28.6	5	819,063	0.6

Abbreviations: No., number; y, years.

^a^Crude incidence rate per 100,000 person-years.

^a^Includes those of American Indian/Alaska Native, Asian/Pacific Islander, and unknown race or ethnicity.

**Figure 1a:**
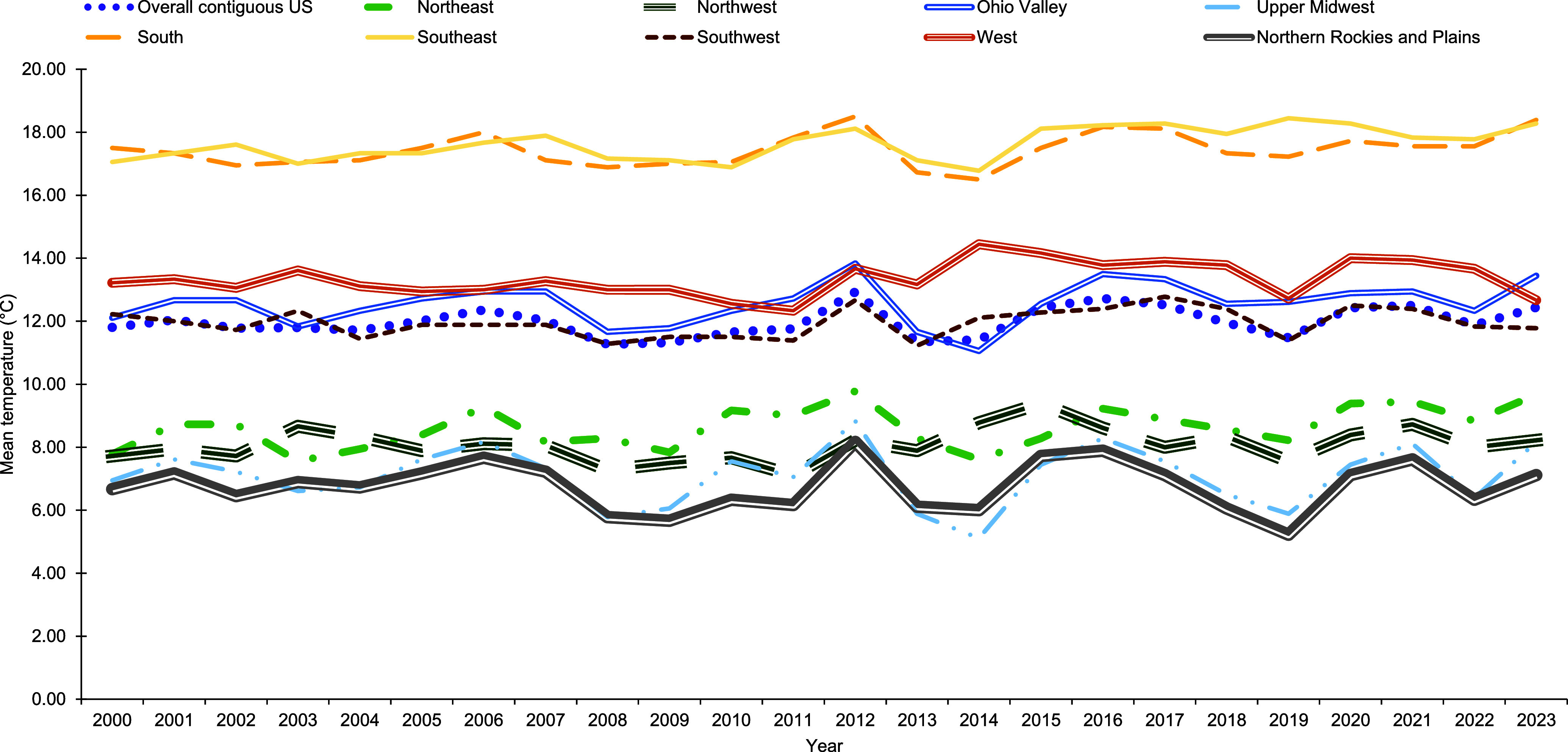
Overall and Region-specific Annual Mean Temperature, Contiguous U.S., 2000–2023

**Figure 1b:**
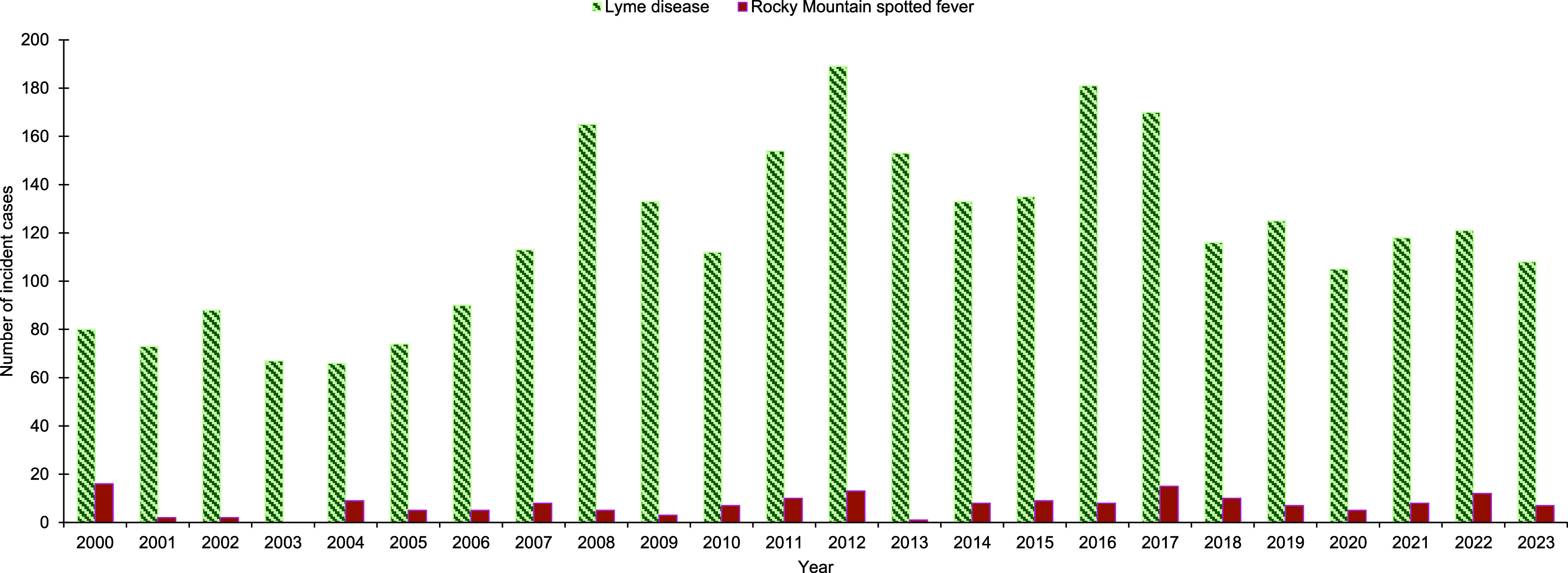
Overall and Region-specific Annual Total Precipitation, Contiguous U.S., 2000–2023

**Figure 3a:**
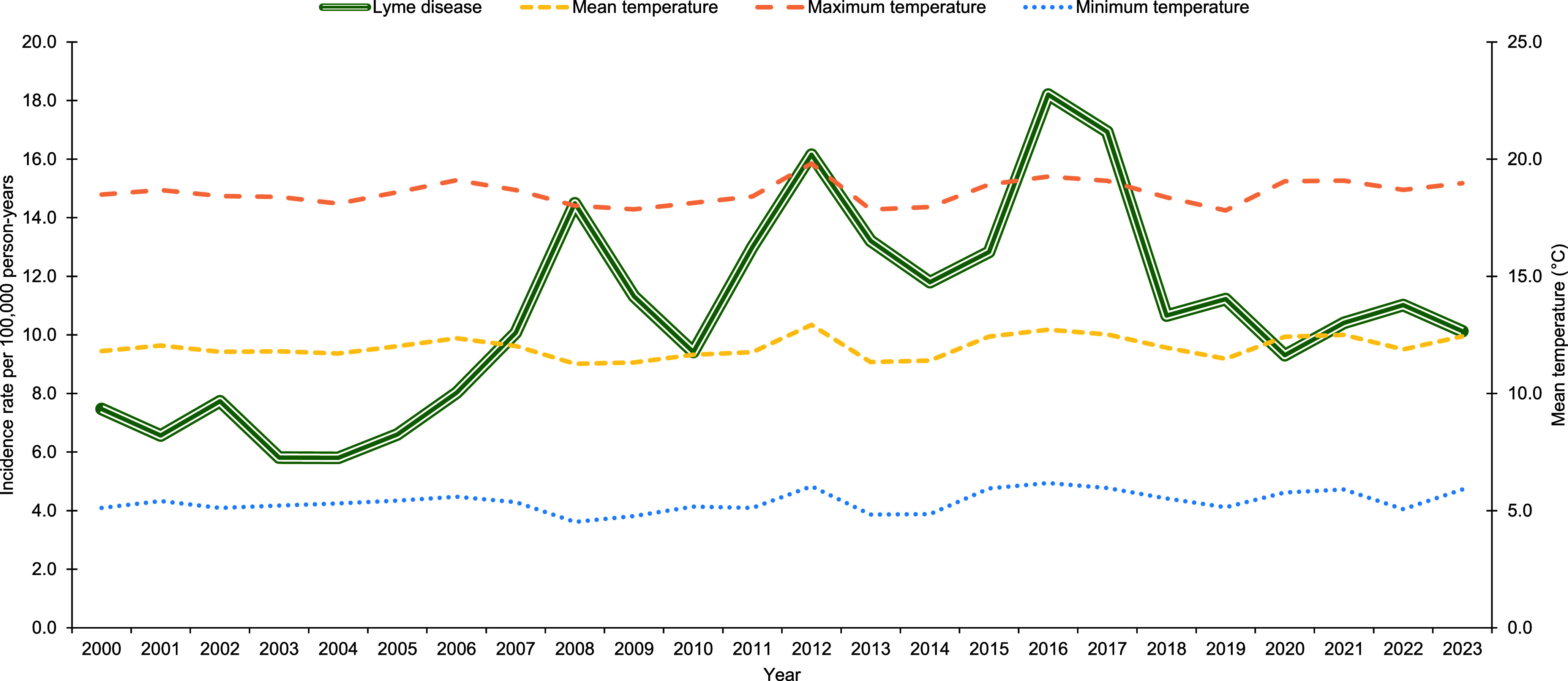
Overall Annual Mean Temperature and Crude Annual Incidence Rate of Lyme Disease, Active Component Service Members, Contiguous U.S., 2000–2023

**Figure 3b:**
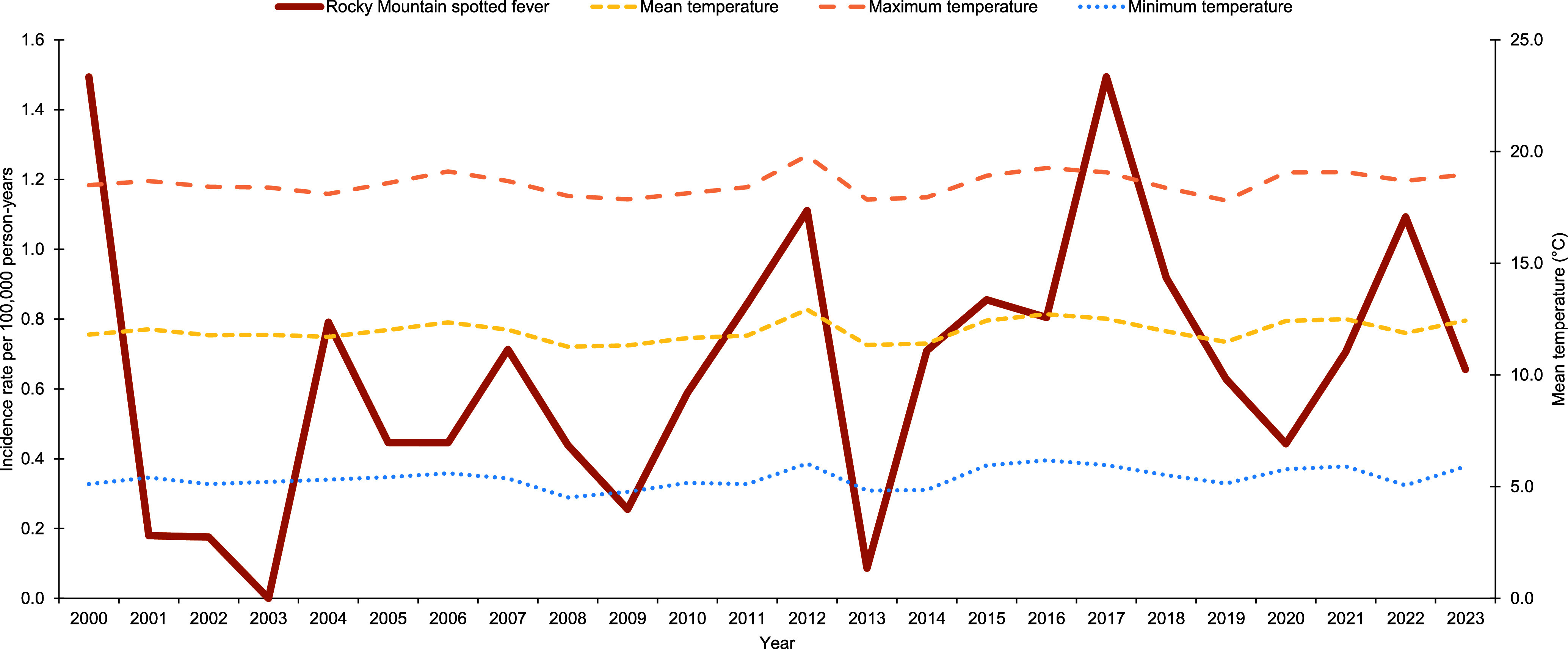
Overall Annual Mean Temperature and Crude Annual Incidence Rate of Rocky Mountain Spotted Fever, Active Component Service Members, Contiguous U.S., 2000–2023

**Figure 4a:**
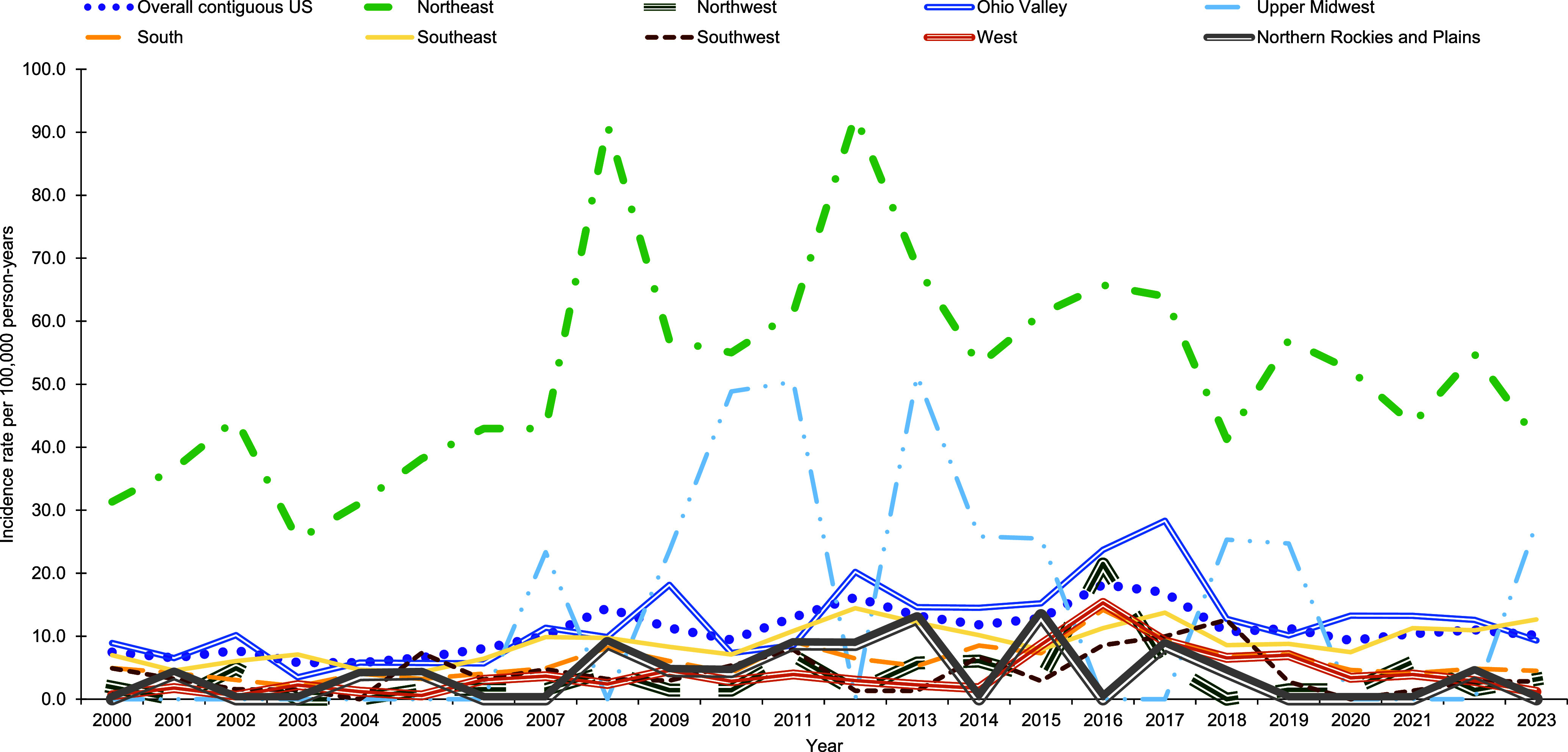
Overall and Region-specific Crude Annual Incidence Rates of Lyme Disease, Active Component Service Members, Contiguous U.S., 2000–2023

**Figure 4b:**
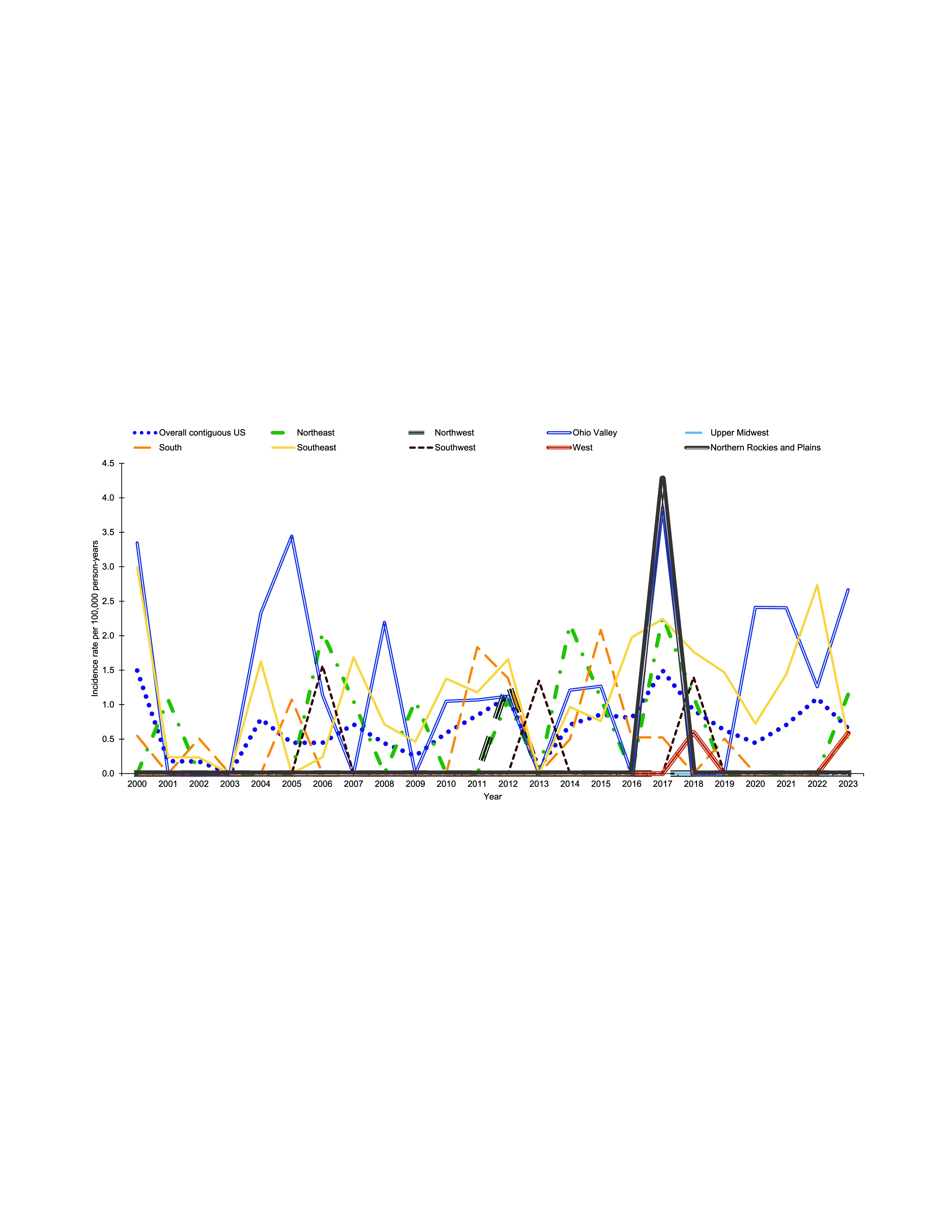
Overall and Region-specific Crude Annual Incidence Rates of Rocky Mountain Spotted Fever, Active Component Service Members, Contiguous U.S., 2000–2023

**Table 2:** Adjusted Incidence Rate Ratios of Lyme Disease and Rocky Mountain Spotted Fever, by Climatic and Demographic Variables, Active Component Service Members, Contiguous U.S., 2000–2023

	Lyme Disease			Rocky Mountain Spotted Fever		
Climactic or Demographic Variable	aIRR^a^	95% LL	95% UL	aIRR^a^	95% LL	95% UL
Annual mean temperature	1.0	1.0	1.1	0.7	0.6	0.9
Annual minimum temperature	0.9	0.9	1.0	1.2	1.0	1.4
Annual maximum temperature	0.9	0.9	1.0	1.0	0.9	1.2
Annual total precipitation	1.0	1.0	1.0	1.0	1.0	1.0
**Regional climate**						
Northeast	Reference	–	–	0.2	0.1	0.3
Southeast	1.5	1.3	1.6	Reference	–	–
Northwest	0.2	0.2	0.3	0.0	0.0	0.1
Ohio Valley	0.7	0.7	0.8	0.4	0.3	0.6
Upper Midwest	0.4	0.3	0.4	0.1	0.0	0.2
South	1.0	0.9	1.2	0.6	0.5	0.7
Southwest	0.4	0.4	0.5	0.1	0.1	0.2
West	0.5	0.4	0.5	0.1	0.1	0.2
Northern Rockies and Plains	0.2	0.2	0.2	0.1	0.0	0.1
**Age group, y**						
<20	Reference	–	–	Reference	–	–
20-24	1.0	1.0	1.0	1.0	0.9	1.2
25-29	1.5	1.5	1.6	1.7	1.6	0.6
30-34	2.1	2.0	2.1	2.5	2.2	2.8
35-39	2.1	2.1	2.2	2.3	2.1	2.6
40+	2.3	2.2	2.4	2.6	2.3	3.0
**Racial and ethnic group**						
White, non-Hispanic	Reference	–	–	Reference	–	–
Black, non-Hispanic	0.4	0.4	0.4	0.4	0.4	0.4
Hispanic	0.6	0.6	0.6	0.5	0.5	0.6
Other/unknown^b^	0.7	0.7	0.7	0.6	0.6	0.7
**Sex**						
Male	Reference	–	–	Reference	–	–
Female	1.5	1.5	1.5	1.0	0.9	0.6
**Service branch**						
Army	Reference	–	–	Reference	–	–
Navy	0.9	0.9	0.9	1.4	1.3	1.5
Marine Corps	1.1	1.0	1.1	3.1	2.8	3.3
Air Force, Space Force	0.7	0.7	0.7	0.9	0.8	1.0
Coast Guard	1.9	1.9	1.9	1.5	1.4	1.8

Abbreviations: aIRR, adjusted incidence rate ratio; LL, lower limit; UL, upper limit; y, years.

^a^Adjusted for annual mean temperature, annual minimum temperature, annual maximum temperature, annual total precipitation, regional climate, age group, racial / ethnic group, sex, and service.

^a^Includes those of American Indian/Alaska Native, Asian/Pacific Islander, and unknown race or ethnicity.

## AUTHORS' AFFILIATION

Defense Health Agency, Armed Forces Health Surveillance Division, Silver Spring, MD: Ms. Denagamage, Dr. Mabila
